# The players may change but the game remains: network analyses of ruminal microbiomes suggest taxonomic differences mask functional similarity

**DOI:** 10.1093/nar/gkv973

**Published:** 2015-09-29

**Authors:** Tasia M. Taxis, Sara Wolff, Sarah J. Gregg, Nicholas O. Minton, Chiqian Zhang, Jingjing Dai, Robert D. Schnabel, Jeremy F. Taylor, Monty S. Kerley, J. Chris Pires, William R. Lamberson, Gavin C. Conant

**Affiliations:** 1 Division of Animal Sciences, University of Missouri, Columbia, MO 65211, USA; 2 Department of Civil & Environmental Engineering, University of Missouri, Columbia, MO 65211, USA; 3 Informatics Institute, University of Missouri, Columbia, MO 65211, USA; 4 Division of Biological Sciences, University of Missouri, Columbia, MO 65211, USA

## Abstract

By mapping translated metagenomic reads to a microbial metabolic network, we show that ruminal ecosystems that are rather dissimilar in their taxonomy can be considerably more similar at the metabolic network level. Using a new network bi-partition approach for linking the microbial network to a bovine metabolic network, we observe that these ruminal metabolic networks exhibit properties consistent with distinct metabolic communities producing similar outputs from common inputs. For instance, the closer in network space that a microbial reaction is to a reaction found in the host, the lower will be the variability of its enzyme copy number across hosts. Similarly, these microbial enzymes that are nearby to host nodes are also higher in copy number than are more distant enzymes. Collectively, these results demonstrate a widely expected pattern that, to our knowledge, has not been explicitly demonstrated in microbial communities: namely that there can exist different community metabolic networks that have the same metabolic inputs and outputs but differ in their internal structure.

## INTRODUCTION

Vertebrates play host to an enormous diversity of microbes, and while the numbers alone are impressive, exceeding our own cell count by at least an order of magnitude (1), it is the role of these organisms in critical host life processes that is both more fascinating and less understood. For instance, in species such as ruminants, the microbes of the digestive system degrade substrates such as cellulose that are inaccessible to the enzymes encoded by the host genome. The result of this symbiosis is to allow these animals to subsist off of diets that would not sustain life in most other vertebrates or invertebrates (2–4). In general, the symbiosis functions by the host animal absorbing volatile fatty acids (VFAs) and amino acids produced by microbes and using these compounds both as energy sources and for biosynthesis (5,6). The microbes that collectively allow for this remarkable capacity are many and varied (7,8), with only a fraction being well characterized and many being completely unstudied (9,10).

Our poor understanding of these organisms is largely due to the fact that they are quite difficult to work with: many or most will not grow in laboratories and they interact not only with the vertebrate host but also with each other in a variety of complex ways. Fortunately, applications of inexpensive DNA sequencing technologies (e.g., metagenomics) are transforming our understanding of the microbial world more rapidly than perhaps any technology since van Leeuwenhoek first looked through his microscope. To date, most surveys of microbiomes both in vertebrate hosts and in other environments have used taxonomic approaches, attempting to identify the different species that are present. The most effective technique for such assays has been using the 16S rDNA gene as a phylogenetic marker (3,7,11–13). This approach has uncovered several interesting associations between the biology of various animal hosts and their microbes: evolutionarily-related organisms have more similar microbiomes that can be explained solely by diet, which is nonetheless an independent predictor of microbiome structure (3,14). Likewise, aberrant phenotypes such as obesity are associated with differences in microbiome taxonomic composition (15).

Metagenomic studies of obesity have also highlighted the importance of linking the taxa present in an environment with broader genetic or functional patterns (16). For instance, Turnbaugh *et* *al*. (15) extended their taxonomic work by also elucidating the metabolic differences between the metagenomes of obese and non-obese individuals. Their results illustrate the rather obvious point that one gene cannot fully capture the complexity of these ecosystems (10,17–20). Indeed, there is considerable evidence that variation in the taxonomic structure of a microbiome may actually mask similarities in the gene content: several studies have found that microbial ecosystems with significant differences in taxonomic composition nonetheless have similar functional categories of genes present (21–23). Likewise, genes from different metagenomic samples of related environments are more similar than are the taxonomic groups found in those same samples (17,24). A single taxonomic catalog also misses processes such as community-structuring rules (species that are always or never found together, as an example) that only become evident in the patterns of taxa co-occurrences (25,26) and changes in community structure over time (27). In both cases, symbiotic relationships not just with the host, but between the microbes themselves are likely critical (28,29).

Gene-centric approaches to the microbiome, while they cannot capture all aspects of this ecosystem complexity, are nonetheless complementary to taxonomic studies. Analyses of the genes present in microbiomes have already helped to identify novel, biotechnologically relevant enzymes (10), to explore how much the microbiome expands the metabolic capacities of the host (18,24) and to associate microbial genes with host diseases (30). Of course, a catalog of genes on its own has many of the same benefits and limitations as does a catalog of organisms. In particular, the presence of common gene functions in microbiomes with different taxonomic composition (21–23) might be attributable the use of high-level annotation categories or the presence of housekeeping genes involved in information processing. However, the advantage of using genic approaches is that this knowledge opens the door for systems biology approaches that can illuminate the connections between those genes (16,19). Metabolism has been a key focus of such gene-centered approaches (10,19,30–31). However, to our knowledge, only a study by Greenblum *et* *al*., (19) has applied the powerful tools of network biology (27,32) to metagenome-scale sequence data.

Here, we apply a similar strategy of mapping reads to an enzyme database as did Greenblum *et* *al*., (19), but in service of a different set of questions. As a result, there are several differences between our approach and theirs: we consider the ecologically complex microbiome of ruminant animals rather than humans, use a more complete reference enzyme database and, most importantly, develop and use a new analysis framework that allows us to explore the ‘metabolic network interface’ between the microbiome and the host. As a result, we introduce a concept of metabolic distance to the microbial networks and use that concept to explore their structures.

In this initial study, we focused on two questions that can be answered even with small samples. First, we asked whether variation at the taxonomic level is invariably matched by similar variation at the metabolic level. By mapping metagenomic reads to both a taxonomic marker (the 16S rDNA gene) and to a catalog of enzymes, we show that some of the observed taxonomic dissimilarities are not necessarily mirrored at the metabolic level. Second, we hypothesized that, while the metabolic inputs and outputs of our samples would be similar (the inputs because of the common diet and the outputs because of the needs of the host species), there would be differences in the internal structure of metabolism between the samples. We tested this hypothesis in several ways, including asking if the variation in copy number for microbial enzymes that can interact with the host is smaller than the variation observed in other enzymes and by showing that the variation in enzyme abundance between the two animals is too large to be explained by sampling variation in the metagenomic sequences. Since the networks are also distinctly non-random in both their reaction and metabolite distributions, we argue that there are metabolically similar but not identical microbial assemblages that are capable of performing the same processes from the host's perspective, even if they appear distinct at the taxonomic level.

## MATERIALS AND METHODS

### Animal selection and DNA sample collection

The two animals used for metagenomic sequencing were selected from a group of Simmental by Angus crossbred steers (*n* = 35; initial body weight = 333.77 ± 8.54 kg; age = 10.14 ± 0.12 mo). These animals were weighed, administered an anthelmintic (1 ml per 50 kg of body weight; Noromectin^®^ Norbrook^®^ Inc., Lenexz, KS, USA) and randomly assigned to four pens (8 steers per pen; 7.6 × 16.5 m) each equipped with two GrowSafe^®^ (Airdrie, AB Canada) feed intake bunks (33). Steers were fed a receiving diet (Supplemental Table S3) for 14 days. At the end of this period, they were transitioned to a concentrate feedlot diet (Supplemental Table S4). Steers were allowed *ad libitum* access to feed and water for the duration of the growth and feed intake trial. For a 120 day period, feed intake was monitored and measured daily by the GrowSafe^®^ feed intake system. Body weights were measured on days 0, 1, 36, 37, 70, 71, 119 and 120 prior to the daily delivery of feed. Feed efficiency was characterized by residual feed intake (RFI) where the model fitted was,

\begin{equation*} \begin{array}{*{20}c} {{\rm Y}_{\rm i} = \beta _0 + \beta _1 \times {\rm daily}\;{\rm body}\;{\rm weight}\;{\rm gain} + } \\ {\beta _{\rm 2} \times {\rm metabolic}\;{\rm mid - test}\;{\rm body}\;{\rm weight}_{\rm i} ,} \\ \end{array} \end{equation*}

where Y_i_ = average actual daily feed intake for animal *i*, β_0_ = regression intercept, β_1_ = partial regression coefficient of actual daily feed intake on daily body weight gain for animal i, and β_2_ = partial regression coefficient of actual daily feed intake on metabolic mid-test body weight for animal *i*. The two steers chosen for the metagenomic experiment had similar body weight gains with different feed efficiencies (Supplemental Table S5). To obtain the microbial DNA for sequencing, we followed the procedure of Guan *et* *al*. (8). Rumen samples from these two animals were taken in the feedlot and were immediately stored on dry ice until returned to the laboratory and were then frozen at −80°C. Particulate matter from the samples was removed by low speed centrifugation. Sterilized zirconia (0.3 g of 0.1 mm) and silicon (0.1 g of 0.5 mm) beads and 1 ml lysis buffer were added to thawed rumen fluid samples and tubes were homogenized using a Mini-Beadbeater-8 at maximum speed for 3 min, incubated at 70°C for 15 min with gentle mixing every 5 min and centrifuged at 4°C for 5 min. The supernatant was transferred to a new 2 ml flat cap tubes and fresh lysis buffer was added to the pelleted beads. The homogenization, incubation and centrifugation were repeated and the supernatants were pooled. Precipitation of nucleic acids, removal of RNA and proteins and purification were completed using the protocol for the QIAamp DNA Stool Mini Kit (Qiagen, Santa Clarita, C, USA).

### Illumina sequencing

Genomic libraries from the two samples (i.e., animals) were constructed following the manufacturer's recommended protocol with reagents supplied in Illumina's DNA sample preparation kit. Genomic DNA was sheared using a BioRuptor (Diagenode, Denville NJ, USA) to generate fragments. The resulting 3′ and 5′ overhangs were removed by an end repair reaction using a 3′ to 5′ exonuclease activity and polymerase activity to blunt the fragment ends. A single adenosine was added to the 3′ ends of the blunt fragment followed by the ligation of Illumina adapters. The adapter-ligated fragments were then size selected using an agarose gel. Fragments of average length 436 (animal 1) or 477 (animal 2) bp were recovered from the gel slice by elution and ethanol precipitation. Each purified library was quantified with a Qubit assay and fragment size was confirmed by Agilent BioAnalyzer High Sensitivity DNA assay.

### Metagenomic sequencing, quality filtering and identification of novel 16S rDNA genes

Libraries were diluted and sequenced according to Illumina's standard sequencing protocol on a GenomeAnalyzer II. The two libraries (one per animal) were each sequenced on two lanes of the instrument, resulting in 150 bp, paired-end sequences. The average insert size was 303 bp for animal 1 and 345 for animal 2. Raw sequence reads are available from NCBI's short read archive (Project accession number PRJNA291523).

Paired-end reads were quality filtered by truncating each read after the first run of three bases with a phred quality score <15 (34). From the filtered reads, any read pair where one or both reads were <100 bases long or had an average quality score of <20 were omitted. These reads were not meaningfully contaminated with DNA from the host animal: only 0.14 and 0.65% of the reads mapped to the bovine genome at 97% identity from animals 1 and 2, respectively. The resulting reads were used for all subsequent analyses (see ‘Results’ section).

### Classification of 16S rDNA-derived reads

To explore the taxonomic structure of these two animals’ ruminal metagenomes, we searched the filtered reads for fragments of 16S rDNA genes. We did so by comparing them to a reference database made up of two pieces: 16S rDNA genes from the Ribosomal Database collection of sequences (35) and 16S rDNA genes from the sequenced prokaryotic genomes available from GenBank (36). To merge these two databases, we purged identical sequences and sequences <1450 bases long or with undetermined nucleotides (e.g., ‘N's). Our final database contained 27 290 sequences. In our previous work (37), we also used the EMIRGE package (38) to identify novel 16S rDNA genes in the samples. However, using EMIRGE or attempting to assemble the sequence reads, while it might uncover further taxonomic diversity in the samples, will also bias the counting of microbial individuals in complex ways that would undermine the comparisons we planned. Direct matching to a sequence database, while subject to some biases (37), is more appropriate.

Database sequences were clustered into Operational Taxonomic Units (OTUs) by first computing all possible pairwise Needleman-Wunsch sequence alignments (39) using our GPU-based pairwise alignment tool (40). From these pairwise alignments, we created a network in which each sequence was a node and edges connected sequences with ≥97% identity. OTUs were defined as connected components within this network. We next used Bowtie (41) to align reads to the database sequences. If both the forward and reverse reads matched sequences from one and only one OTU with ≥ 97% identity, we classified that pair as representing an individual from that OTU. These stringent criteria resulted in a relatively small number of microbial individuals being assigned to OTUs: nonetheless, there were 18 OTUs with more than five individuals identified in both animals, suggesting that we have reasonable sample depth for our analyses.

**Figure 1.**
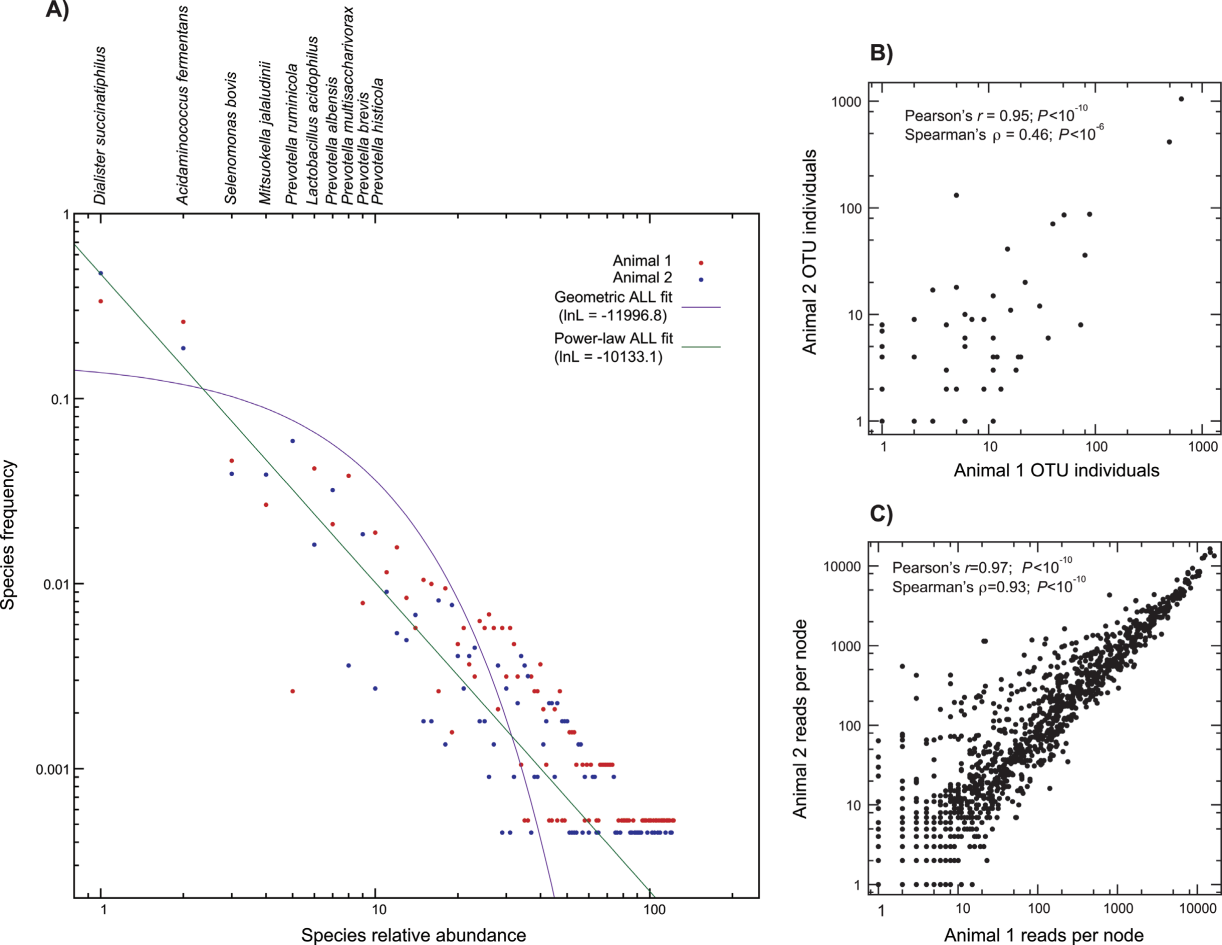
Taxonomic and functional analyses of the ruminal metagenomes of two bovine individuals. (**A**) Distribution of the 122 OTUs found, with models of the abundance distributions of those OTUs. On the *x*-axis is the rank abundance of each OTU for animal 1 (red) and animal 2 (blue): the most abundant OTU has rank 1 and so forth. On the *y*-axis is the proportion of that animal's total microbial individuals that that OTU comprises. We fit two statistical distributions to these data: a discrete power-law (green) and a geometric (purple; see ‘Materials and Methods’ section). Taxon names for the first 10 OTUs are shown above the panel. (**B**) Correlation between OTU abundance for animal 1 (*x*-axis) and animal 2 (*y*-axis). Note the log scale in both cases. (**C**) Correlation of the number of mapped read pairs for each metabolic reaction (node) for the two animals. Axes are as for B and are also shown in log-scale.

As shown in Figure 1, we next fit the OTU counts to both a power-law and a geometric distribution (37). We computed *L_b_*, the likelihood obtained from fitting both animals to a common distribution, and *L_1_* and *L_2_*, those obtained when allowing distinct distributions for each animal. We then compared the value Δln*L* = (ln(*L_1_*) + ln(*L_2_*)) *−* ln(*L_b_*) from the real samples to the same value computed on 1000 datasets where the OTUs were randomly repartitioned between the two animals (37). As the Δln*L* estimated from the actual dataset was always greater than the values seen in randomized samples, we could reject the null hypothesis of a common OTU distribution for the two animals (*P* < 0.001).

### Extraction of a reference metabolic enzyme database for metagenomics

We obtained the complete catalog of microbial metabolic networks known to the MetaCyc project (42). This catalog includes metabolic reconstructions for more than 2000 microbes. Each reconstruction includes a set of reactions inferred to be possible in that organism, with substrates and products for each. Some, or all, of these reactions are annotated to enzyme sequences that are also included with the reconstruction. We thus created an enzyme database from MetaCyc where each enzyme sequence was linked to one or more metabolic reactions.

### Host and microbial metabolic network construction

**Figure 2.**
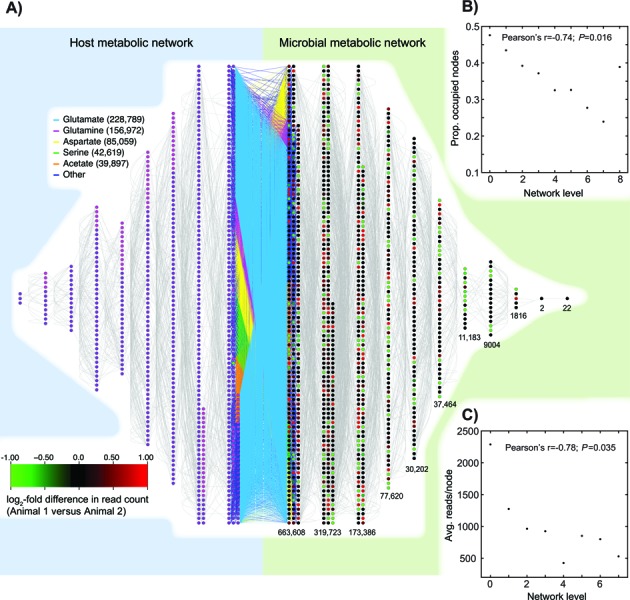
Structure of the metabolic network induced by the microbiome of the bovine rumen. (**A**) Interface of the host (left) and microbial (right) metabolite networks. Nodes (circles) are reactions: two nodes are linked by an edge if they share a metabolite (gray lines, currency metabolites were excluded at a stringency of 25—e.g., *N_25_* see ‘Materials and Methods’ section). The interface of the two networks is illustrated in the center. The five most common interface metabolites are shown in color; all other interface metabolites are blue. Host/microbial reactions that employ an interface metabolite are shown as the two closest layers: subsequent layers are removed from this interface based on their nearest metabolites to that interface (‘Results’ section). Microbial nodes are color-coded based on the log_2_ difference in the normalized number of read pairs mapped for animal 1 versus animal 2: thus green nodes represent over-abundance in animal 2 and red ones in animal 1. Only nodes with a binomial probability of *P* ≤ 0.01 of having an equal numbers of reads are shown with fold-differences (all others shown in black). Numbers under each layer give the total number of read pairs mapped to that layer. Host nodes are shown in purple (reactions annotated in humans), magenta (reactions annotated in cattle) and green (the added pseudo-reaction that allows the use of butyrate; ‘Materials and Methods’ section). (**B**) The proportion of nodes in each microbial layer with a mapped read pair is inversely correlated with the distance from the host metabolic network (see ‘Results’ section). (**C**) The number of mapped read pairs per node is also inversely correlated with the distance to the host metabolic network.

We next constructed two metabolic networks for our analysis, one for the bovine host and one for the microbes. In our framework, nodes in the metabolic network (circles in Figure 2A) are reactions from MetaCyc. For the microbial network, we merged any two reactions with identical metabolite lists, resulting in 6140 microbial nodes with at least one annotated enzyme sequence.

For the host metabolic network, we combined the human and bovine MetaCyc metabolic reconstructions. First, using our previously described approach (43,44), we inferred the bovine orthologs of human MetaCyc enzymes using release 75 of Ensembl (45). Of the 2863 human enzyme-coding genes annotated in MetaCyc and also found in Ensembl release 75, we identified 1:1 bovine orthologs for 2553 (89%). These genes corresponded to 1850 network nodes. We then added to these reactions all bovine reactions not also in the human reconstruction (the bovine metabolic network is smaller than the human reconstruction, with only 1404 annotated reactions, which is the reason for using the human network as an initial framework). The resulting host metabolic network had 2126 nodes.

For all networks, we defined edges in the metabolic networks to connect pairs of nodes that share a metabolite. Only annotated metabolites present in at least two taxa were considered for these purposes. Because a handful of metabolites, such as water and hydrogen ions, occur in an enormous number of reactions, it is necessary to remove these ‘currency metabolites’ from the metabolic networks. The alternative would be to have nearly every reaction connected to every other reaction. Unfortunately, there is no universal definition of a currency metabolite (46). Instead, we use three definitions of currency metabolites: namely compounds that appeared in more than 25, more than 50, or more than 100 MetaCyc reactions (47). Each definition corresponds to one metabolic network, which we refer to as *N_25_, N_50_* and *N_100_*, respectively. The number of currency metabolites defined ranged from 261 (for *N_25_*) to 174 (for *N_100_*).

### Defining the interface between the host and microbial metabolic networks

**Figure 3.**
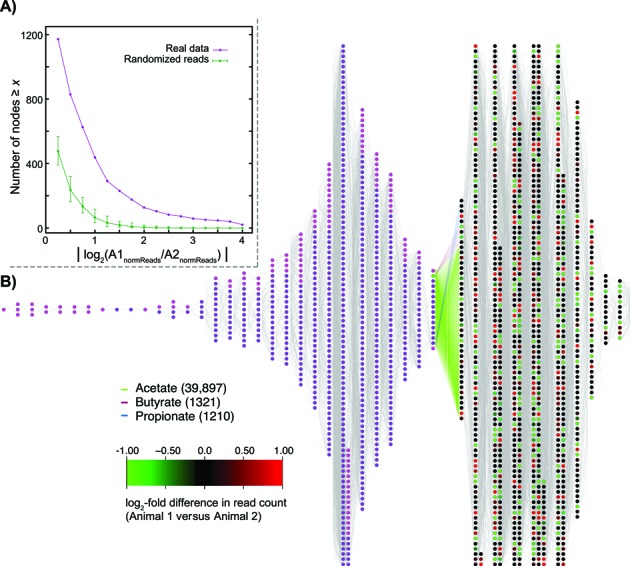
Node-level differences between animals and volatile fatty acid metabolism. (**A**) The two animals studied differ considerably the relative abundance of many enzyme-coding genes. On the *x*-axis is the absolute value of the log_2_ difference in enzyme abundance for the two animals, after correcting for the different number of reads obtained for the two animals. Hence, a value of 2 on this axis represents a four-fold excess of reads mapping to a particular node either in animal 1 over animal 2 relative to the other animal. On the *y*-axis is the number of nodes seen to have a log_2_ abundance difference at least as large as *x* (purple curve, real data). For comparison, we show the number of such nodes seen when we randomly reassign reads to animals 1000 times (green curve; error bars represent the maximum and minimum numbers seen across all 1000 randomizations. (**B**) Enzymes involving the volatile fatty acids (VFAs) represent a minor fraction of the metagenomic reads. Host/microbe merged metabolic networks when only the three VFAs were considered as interface metabolites (*N_50_*). All other details are as for Figure 2A. Note that the network is much smaller (fewer reactions are linked between the host and the microbes). Moreover, even these linkages are weaker, since the number of mapped reads to the distance 0 microbial nodes is much lower than in Figure 2A—glutamate alone accounts for roughly four times as many reads as are present in the entire ‘layer’ for this network.

We defined three potential sets of metabolites exchanged between the microbes and the host animal, sets that we refer to as ‘interface metabolites’. The most minimal set (set VFA) consisted of (the anions of) three VFAs: acetate, propionate, and butyrate. VFAs are important nutrients for ruminants (48) but unfortunately are somewhat poorly represented in MetaCyc. Thus, two VFAs (valerate and isovalerate) are absent entirely from MetaCyc and isobutyrate, while present, cannot be mapped to any vertebrate reactions (e.g., there is no reaction in MetaCyc converting isobutyrate to the vertebrate metabolite isobutyryl-CoA). Even butyrate itself does not have a corresponding vertebrate reaction, but we added a pseudo-reaction (known from the microbes) to the vertebrate network that converts butyrate to butyryl-CoA (green node in Figures 2 and 3).

Our second set (VFA + AA) expands the VFA set to include the 20 amino acids, which are also important ruminant nutrients. Finally for completeness, we created a greatly expanded interface metabolite set (VFA + AA + HUM). In addition to the metabolites of VFA + AA, this set incorporates a large number of metabolites known to be transferred into human cells from the extracellular matrix. To obtain this set, we started with the list of 404 exchangeable human metabolites from the human metabolic network of Duarte et al. (49). We matched 234 of these compounds to their corresponding entries in MetaCyc (most of the unmatched compounds are complex eukaryotic polysaccharides or lipopolysaccharides; data not shown). From those compounds, we selected only the organic compounds and excluded the DNA/RNA nucleotides, carbon dioxide and NAD and NADP, yielding a final list of 204 compounds (Supplemental Table S2).

Given the interface metabolite list and these added reactions, we defined edges between the host and microbial metabolic networks as any case where a reaction in the host network and one in the microbe network shared an interface metabolite. The result was a single merged metabolic network for the host and microbes (VFA + AA shown; Figure 2A).

### A distance-based approach to structuring the merged metabolic networks

Using the merged metabolic network, we calculated the distance between each reaction in the host or microbial sub-network and the other sub-network. To do so, we calculated the minimum path length between every pair of nodes using Dijkstra's algorithm (50). We then defined the distance between a node in one sub-network and the other sub-network as the number of intermediate nodes that must be crossed in order to reach the nearest node in the other sub-network. These integer distances define the layers shown in Figure 2A; a host node and a microbial node that share an interface metabolite have the minimal distance of 0.

### Translating metagenomic reads

We next assessed the status of the microbial metabolic network in each animal using the metagenomic data. To do so, for each paired read, we computed all six frame-translations and retained any translations for which both reads in the pair had open reading frames (ORFs) longer than 45 amino acids. We then used these metagenomic ORFs as queries in searching an enzyme database (see next section).

### Nucleotide-based searches of the enzyme database are inefficient

We queried 1000 reads whose translations produced strong hits to the MetaCyc protein database (>80% amino acid identity, see ‘Results’ section) against the nucleotide version of that database. The results were discouraging; only 30% of those reads produced any BLASTN (51) hit.

### Searching translated reads against the enzyme database

Programs such as BLAST, because they are optimized to not miss distant matches, are very slow for searching metagenomic ORFs against protein databases. To speed our searches, we developed a tool based on the SeqAn library and OpenMP (52). For each read, the tool first searches for pairs of identical seven amino acid ‘seed’ matches between the read and the database sequences. Any read/database sequence pair possessing two such seed hits is then exactly locally aligned using the standard Smith–Waterman approach (53). If the resulting local alignment demonstrates 80% amino acid identity over 80% of the ORF, it is reported as a match. To reduce the size of the database searched, we first used our search tool on the database itself, removing sequences with greater than 97% amino acid identity to other database sequences over 97% of their length. The resulting database had roughly 760 000 enzyme sequences.

We next assigned reads to metabolic network nodes if they had hits to annotated sequences for that node meeting the above criteria. Enzymes in the sequence database are often annotated as catalyzing more than one reaction. In such cases, if an ORF pair has hits to multiple database sequences, one of three scenarios can be observed. First, all of the database hits may map to the same set of reactions. Second, some of the database hits may only map to a subset of the reactions of other hits. Finally, different database hits may map to non-overlapping sets of reactions. In the first two cases, we compute the reactions catalyzed by an ORF pair as the union of the reactions catalyzed by each enzyme with hits to that ORF pair. In the last case, the read pair is discarded because we cannot be certain of the identity of the reactions in question. Cases where read pairs did not both map to the same enzymes were likewise omitted. Note that while this mapping approach is applicable to many metagenomic datasets, our analysis of differences between animals from similar environments precludes comparing these data to other samples from different species (37) or environments (10). Correlation statistics reported in ‘Results’ were calculated in R (54).

We sought to assess if there were highly connected enzymes from MetaCyc that might be expected to be in the rumen that were not identified with our read mapping approach. Supplemental Table S6 lists the 10 most connected reactions (e.g., nodes with the highest number of edges) from networks *N_25_, N_50_* and *N_100_* with no mapped reads. No major omissions are apparent from these data.

### Correlation of number of occupied nodes or number of mapped reads and network layer

For each layer of the microbial network in Figure 2A, we computed the average proportion of nodes in that layer with mapped reads (*p*) and the average number of reads mapped to those nodes with at least one mapped read (*r*). We then calculated Pearson's correlation coefficient between these two values and the layer's distance to the host network. To assess whether the observed trends were stronger than expected by chance we randomized the read assignments among the microbial nodes 1000 times and recalculated these two correlations. For *p*, we randomized read counts across all microbial nodes, occupied or not. For *r* (the average number of mapped reads), we only randomized the read numbers within the set of nodes with at least one mapped node.

### Testing abundance differences between the two animals

Sampling effects will lead to there being some nodes with differing numbers of mapped reads for each animal. To see if the observed differences in read counts for the nodes could be explained by such sampling variation, we first computed, for a range of abundance differences, the number of nodes with such an abundance difference in the metagenomic data. Because we obtained differing numbers of total reads that mapped to MetaCyc nodes for the two animals, before computing the log_2_ ratio of abundances for the animals, we normalized all reads counts by the total number of reads mapped for each animal (*n_1_* and *n_2_*; respectively). We then pooled the mapped reads, randomly reassigned *n_1_* of them to animal 1 and *n_2_* of them to animal 2 and recomputed the number of nodes with every abundance difference (see Figure 3A). Repeating this procedure 1000 times allowed us to assess if the number of differentially abundant nodes for the two animals could be explained by chance.

## RESULTS

### Metagenomic sequencing of rumen fluid from two steers

We sampled ruminal fluid from two steers, extracting microbial DNA from that fluid and then shotgun sequenced the DNA on an Illumina GenomeAnalyzer II. The result was 33.7 million paired reads for animal 1 and 30.9 million paired reads for animal 2. Mean read length after quality trimming was 134.5 bp for animal 1 and 135.8 bp for animal 2 (‘Materials and Methods’ section).

### rDNA analysis

To define microbial OTUs, we computed those OTUs (‘Materials and Methods’ section) from a database of known 16S rDNA sequences by all-against-all pairwise alignment and sequence clustering (40). Using this common set of OTUs, we mapped the shotgun metagenomic reads from the two animals onto that database: any read pair that mapped to sequences from one and only one OTU was assumed to represent an instance of that OTU (37). A total of 4127 read pairs mapping to 16S rDNA genes, comprising 122 distinct OTUs (56 common to both animals), were identified in the two samples (Figure 1A). Supplemental Table S1 gives a phylum-level breakdown of the identified taxa. To statistically describe these OTU counts, we fit the OTU rank abundance distribution to both a power-law and a geometric distribution. In so doing, we found the power-law distribution to provide a statistically better fit (ln-likelihood = −10 133 versus −11 997 for the geometric distribution). From this result, we conclude that the rumen environment has a highly uneven taxonomic distribution dominated by a handful of OTUs but with many other rare OTUs also present. The OTU distributions were not identical for the two animals: allowing each to have its own power-law or geometric OTU distribution significantly improved the fit (*P* < 0.001, ‘Materials and Methods’ section). We also normalized the read counts for each animal and compared the percentage of reads from the two that were mapped to the same OTU: only 44% of reads from the two animals mapped to the same OTUs.

### Metabolic networks

It is conceptually obvious that one could infer the gene contents of a metagenome from a reference database. As we were interested in metabolism, we chose the collection of metabolic network reconstructions in MetaCyc (42) as our database. Unfortunately, nucleotide sequences are too degenerate for this approach (‘Materials and Methods’ section). We therefore instead employed our new, faster and more sensitive amino acid-based pipeline to search for sequence similarity between the open reading frames (ORFs) found in the reads and the enzymes of MetaCyc. First, we computed six-frame translations of the metagenomic reads and retained long ORFs. Using a custom sequence similarity search tool (‘Materials and Methods’ section), we then matched these ORFs to MetaCyc's enzyme sequence database (42). Also using that database, we defined a community metabolic network (19) for the microbes, where nodes are enzyme-catalyzed reactions (represented by one or more protein sequences from MetaCyc) and edges connect reactions that share a metabolite (Figure 2A). Currency metabolites (such as water and adenosine triphosphate) were excluded from all networks at three stringencies from high (*N_25_*) to moderate (*N_100_*; ‘Materials and Methods’ section). While these networks disregard the compartmentalization of the ruminal ecosystem into distinct cells, any compartmentalized metabolic network for this environment will be a subset of the community network.

More than 1.2 million read pairs from the two animals mapped uniquely to protein sequences from MetaCyc. Those protein sequences in turn catalyze one or several reactions each (‘Materials and Methods’ section). Of the 6140 nodes in the microbial metabolic network, 2022 nodes had at least one mapped read pair and 1734 had read pairs mapped from both animals. A reaction from the electron transport chain interconverting ubiquinones and ubiquinols had the largest number of mapped reads: 16 443 reads from a single animal.

We were concerned that the number of reads that mapped to a reaction might simply be a function of the number of known enzyme sequences for that reaction. For those nodes with at least one read mapped, there is a statistically significant correlation between the number of reads that mapped to a reaction and the number of known enzyme sequences for that reaction (Pearson's *r =* 0.31, Spearman's ρ = 0.57, *P* < 10^−10^ in both cases). However, given that one strong predictor of a reaction having many sequences in the MetaCyc database is that the reaction is widely distributed phylogenetically, we argue that the magnitude of these correlations do not suggest database coverage is the primary signal being detected here. Similarly, the number of reads mapped to a node is correlated to that node's metabolic centrality (node degree), but this effect is rather weak (0.15 < Pearson's *r* < 0.17, 0.18 < Spearman's ρ < 0.24 across *N_25_, N_50_* and *N_100_*; *P <* 10^−5^). Central nodes also have a very slight tendency to be more variable between animals after accounting for the number of mapped reads for networks ­*N_50_* and *N_100_* (Pearson's partial correlation of node degree and the *P-*value of the test of equal read abundance: −0.08; *P <* 0.006), but no such effect was seen for *N_25_*.

The two animals are much more similar in their enzyme node profiles than in their OTUs: 74% of the reads between the two animals matched at the node level. A similar situation was observed when considering the between-animal correlation in OTU counts or in nodes with mapped reads: the OTU mapping was more variable between individuals than was the node mapping, especially when considering non-parametric correlation statistics (Figure 1B versus C). Of course, the smaller sample of OTU individuals relative to enzyme genes will induce increased OTU variance through sampling. However, note that even the two most abundant OTUs differ in frequency between the samples, despite the fact that hundreds of individuals of both were identified in each sample (*P* < 10^−5^; χ^2^ tests). Moreover, there are 2022 reactions with mapped sequence reads compared with 122 OTUs detected, meaning that the metabolic network in fact has more potential points of variation.

### Metabolic interaction between the host and microbiome

We next sought to assess how the microbiome might interact metabolically with the bovine host by inferring a host metabolic network. To do so, we first used our previously described orthology inference tool (43) to map the human metabolic network onto the bovine genome. We then merged that network with the bovine metabolic network from MetaCyc to create our final estimated bovine metabolic network (‘Materials and Methods’ section). We linked the resulting host network to the microbial network using 23 *interface metabolites:* three VFAs and the twenty amino acids (VFA + AA; ‘Materials and Methods’ section). These interface metabolites allowed us to infer the layered networks of Figures 2 and 3. Supplemental Figure S1 illustrates the network of Figure 2A with nodes colored by the number of mapped reads.

We used these merged networks to measure how closely a given microbial reaction interacts with the host's metabolism. A microbial reaction that involves one of the interface metabolites has distance 0 from the host (and vice versa for host reactions). If a reaction involves a metabolite that is also involved in one of the distance 0 reactions (but does not itself possess an interface metabolite), that reaction has distance 1, and so forth. Note that interface metabolites were considered whether or not they were also defined as currency metabolites (see above). All of our observations also hold for a larger set of interface metabolites including the VFAs, the amino acids and human extracellular metabolites (data not shown; set VFA + AA + HUM; ‘Materials and Methods’ section). Supplemental Figure S2 illustrates how the network structure changes using this larger interface set.

This layered network structure has several features that suggest that it reflects an underlying biological reality. There is a strong correlation between the distance to the host network (layer number) and the proportion of nodes with at least one mapped read pair (*p*) and the average number of reads that mapped to a node (*r*) for all three networks (Table 1 and Figure 2B and C). Since the total set of nodes in the network represents all the reactions that are currently known, it is not unexpected that only a subset of those reactions would be used in the restricted environment of the rumen. There is also generally less variability in read count between the two animals for the nodes from the closest layer than from the next two, although this trend is not always statistically significant (Table 2). One might think that these associations are simply the product of more known sequences for reactions that are closer to the host metabolic network. However, there is no correlation between a node's layer number and the total number of known sequences for that node (−0.06 < Pearson's *r* < −0.04; −0.05 < Spearman's *ρ* < −0.03 across *N_25_, N_50_* and *N_100_*).

**Table 1.** Association of layer position and microbiome structure

Network^a^	Correl. (*p, d*)^b^	*P*^c^	Correl. (*r, d*)^d^	*P*^c^
*N_25_*	−0.74	**0.03**	−0.78	**0.026**
*N_50_*	−0.84	**0.007**	−0.94	**0.007**
*N_100_*	−0.67	0.07	−0.98	**<0.001**

^a^Three different metabolic networks based on the exclusion of currency metabolites: *N_25_* implies that all metabolites participating in >25 or more reactions were considered currency metabolites and were ignored in network construction (‘Materials and Methods’ section). Layer structure was inferred with the VFAs and amino acids (set VFA + AA) used as the interface compounds.

^b^Pearson's correlation of the proportion a layer's microbial reactions/nodes occupied by metagenomic read pairs (*p*) and the layer number (*d* or distance of the microbial reaction to the host network). Only a continuous set of layers with at least 10 nodes each were considered (e.g., we stopped considering layers after the first one with <10 nodes).

^c^*P*-value for the test of the hypothesis of a stronger negative correlation between distance (*d*) and either *p* or *r* than expected. We randomized the read locations in the network and recalculated *p* and *r* 1000 times and again calculated the correlation coefficient, counting how often it was as small or smaller than that seen in the actual data (one column left; see ‘Materials and Methods’ section). Values shown in bold are significant at *P<*0.05.

^d^Pearson's correlation of the average number of read pairs mapped to each node (*r*) and the layer number (*d* or distance of the microbial reaction to the host network). Only nodes occupied by at least one read pair were considered. Also, only a continuous set of layers with at least 10 nodes each were considered (e.g., we stopped considering layers after the first one with <10 nodes).

**Table 2.** Association of layer position and animal-to-animal enzyme variation

Network^a^	Binomial cutoff^b^	Avg log_2_(A_1_/A_2_)_0_ − Avg log_2_(A_1_/A_2_)_1_^c^	*P*^d^	Avg log_2_(A_1_/A_2_)_0_ − Avg log_2_(A_1_/A_2_)_2_^c^	*P*^d^
*N_25_*	0.05	−0.22	**0.003**	−0.28	**0.03**
	0.01	−0.34	**0.006**	−0.24	0.06
	0.001	−0.36	**0.015**	−0.24	0.09
	0.0001	−0.46	**<0.001**	−0.32	0.05
*N_50_*	0.05	−0.36	**<0.001**	−0.24	**0.03**
	0.01	−0.41	**0.001**	−0.25	**0.03**
	0.001	−0.43	**0.001**	−0.24	0.05
	0.0001	−0.53	**<0.001**	−0.29	**0.03**
*N_100_*	0.05	−0.31	**0.002**	−0.24	**0.02**
	0.01	−0.36	**0.002**	−0.25	**0.04**
	0.001	−0.36	**0.007**	−0.24	0.05
	0.0001	−0.46	**<0.001**	−0.29	**0.03**

^a^Three different metabolic networks based on the exclusion of currency metabolites: *N_25_* implies that all metabolites participating in >25 or more reactions were considered currency metabolites and were ignored for network construction (see ‘Materials and Methods’). Layer structure was inferred with the VFAs and amino acids used as the interface compounds (set VFA + AA).

^b^Binomial cutoff for determining if the two animals differ significantly in the number of reads for a given node. We computed the proportion *p_1_* of the total metagenomic read pairs belonging to animal 1 and then asked, for each node, whether there were significantly fewer or more read pairs mapped to that node from animal 1, given *p_1_* under a binomial distribution. Four cutoff values for significant differences in mapped read pairs were used.

^c^For each node with a significant difference in read pair count between animals (see previous column), we calculated log_2_(A_1_/A_2_): the log_2 ­_of the ratio of the read counts for the two animals. We then calculated the average of this value for layer 0 and subtracted from it that average for layer 1 (or 2). Hence, the negative values show indicate more variability in mapped read pair counts in layer 1 (or 2) than in layer 0.

^d^*P*-value for the test of the hypothesis of more variable mapped read pair counts in layer 1 (or 2) than in layer 0. We randomized the mapped read paired locations in the network (including nodes with no mapped reads) and recalculated the average differences (previous column) 1000 times, counting how often it was a larger negative value than that seen in the actual data (one column left; see ‘Materials and Methods’ section). Values shown in bold are significant at P<0.05.

### The two sampled animals differ significantly in their distribution of enzyme abundance

Figure 1B and C suggest that the two surveyed animals differ less in their enzyme complements than in their OTU abundances. However, the two animals still differ meaningfully in their metabolic networks. In Figure 3A, we show the number of nodes with differing abundances for the two animals at varying stringencies, compared to the number of nodes with such differences observed when our metagenomic reads are randomly reassigned between the two animals. For a wide range of abundance differences, the real data show much higher divergences between the animals in read abundance than can be explained by chance (*P* < 0.001; ‘Materials and Methods’ section).

### Characterizing the metabolite profile of the microbiome

We can also make limited inferences regarding the set of metabolites that are present in the microbiome by assessing the proteins that are found there and the reactions that they catalyze. We first ranked the interface metabolites by the number of read pairs that mapped to each microbial reaction that involved such an interface metabolite. As can be seen in Figure 2A, glutamate and glutamine are the interface metabolites that were found in reactions with the largest number of mapped read pairs, followed by aspartate and serine.

The total set of metabolites inferred to be present in the microbiome (based on the enzymes present) is also non-random. The number of metabolites associated to nodes with mapped read pairs is smaller than would be expected if the same number of nodes were selected at random (*P* < 0.001 for all three networks). Moreover, the two animals were more similar in their metabolite profiles than would be expected by chance: for all reactions using each metabolite, we computed the net difference in normalized read count between animals. The sum of this statistic across all metabolites was much smaller than that found when the read counts were randomized between the two animals (*P* < 0.001 for all three networks: only nodes with mapped reads were considered). Table 3 lists all compounds where more than 40 000 read pairs were mapped to reactions employing that compound.

**Table 3.** Compounds involved in reactions that have >40 000 read pairs mapped

Compound	Mapped reads	Compound	Mapped reads	Compound	Mapped reads
H^+^	1 342 371	Glutamine	156 972	Aspartate	85 059
ATP	633 063	NADH	154 573	GDP	79 945
Water	612 030	NH_3_	142 055	Fumarate	67 572
Inorg. Phosphate.	438 632	NH_4_^+^	127 611	Acetyl-CoA	48 955
AMP	385 809	Oxaloacetate	121 016	UMP	43 620
ADP	246 006	NADPH	108 199	Formate	43 007
CO_2_	235 605	NADP^+^	108 196	Serine	42 619
Glutamate	228 789	α-ketoglutarate	104 468	Maltotetraose	42 431
Glucose-1-phosphate	166 257	GTP	95 572	Fructose-6-phosphate	41 805
NAD^+^	163 449	Pyruvate	91 701		
Phosphoenolpyruvate	157 805	Coenzyme A	90 809		

### Metabolic networks of volatile fatty acids

Considering that much of ruminal nutrition is thought to be provided by VFAs (48), we next visualized the metabolic interface considering only the three VFAs as the interface metabolites (Figure 3B). Since the amounts of carbohydrate absorbed from the rumen are very low, VFAs provide both the majority of the energy and many biosynthetic precursors for these animals (6). Acetete is the most commonly implicated VFA from the metagenomic reads, just as it is the dominant VFA end-product in the rumen (5). However, as acetate is also a common metabolic intermediate, some caution is warranted in interpreting this result. We were initially surprised to observe that only 2521 microbial read pairs mapped to reactions producing the VFAs butyrate and propionate. However, this figure is actually a 1.8-fold over-representation relative to the database (2521 reads out of 1.2 million mapped, versus 3487 sequences for these reactions from a total of 3.0 million in the MetaCyc database). Recall that we have assayed the gene content, not the gene expression, of this ecosystem: the proposal that 1 in 500 of the different ‘types’ of reactions present in the rumen is involved in terminal VFA production does not appear unreasonable. (Note that we are only counting reactions that actually involve these two metabolites, not all the reactions in the pathways to their synthesis.) In fact, if we assume that the average microbial metabolic network contains about 500 reactions per species, this would imply that each individual in our community has one reaction involving one of these two metabolites. Such a value is reasonably high, since in fact many taxa do not produce these compounds, which are not commonly part of intermediary metabolism.

## DISCUSSION

We have presented an example where two bovine ruminal microbiomes that appeared rather different at the taxonomic level were much more similar in their metabolisms. While we do not, on the basis of two animals, claim this observation is universally true, it is in keeping with the known complexity of microbial ecosystems, where it is both possible to create taxonomically distinct assemblages with similar metabolic properties and taxonomically similar ones with differing metabolic properties (24,55). It also refines previous analyses suggesting that microbial ecosystems can be functionally similar while having distinct taxonomic structures (21–23). By having shown a detailed metabolic example of differing taxa with similar roles, we have begun to uncover the genomic and metabolic sources of such distinctions. Of course, the metagenome does not perfectly reflect the metabolic activities of the microbes present: for instance our study did not assess the effects of gene expression, accounting for which would probably tend to increase the degree of divergence between the two animals (23).

While previous work has compared taxonomy and gene complements (15,17,21–24), genes such as information processing genes may be common across samples without implying ecosystem-level similarities. A metabolic network approach more closely tracks ecosystem-level properties and provides evidence for the proposition that different taxa might have similar functional rules. An alternative explanation is that while housekeeping enzymes are common across taxa (though see 56), taxonomic variation would drive presence/absence differences in key enzymes. Our data do not support this alternative, however: the largest number of reads that mapped to a node in one animal for which no reads were mapped in the other was 101 (out of more than a million total mapped reads). Instead, on average, such differentially present enzymes have only 3.4 reads mapped in one animal compared to zero in the other. Rather it appears that, while drawing on a generally common set of enzymes, the two animals are much more different in their distribution of those enzymes than can be explained by mere sampling variation in the metagenomic data (Figure 3A). This variation is spread throughout the network (Figure 2A); while the animals are actually more different in the number of mapped reads for central reactions, this effect is quite weak. Instead, the networks show coherent patterns that appear to derive from organized interactions with the host animal. Thus, the interface layer of Figure 2A is less variable between animals than are the next two layers (Table 2) and the reactions used by the microbiome decline as a fraction of the possible reactions the further one moves from that interface layer. Collectively, these observations are consistent with the idea that the host environment selects for microbial metabolic networks with a common interface but can tolerate variation in other parts of the network as long as that interface is maintained. Such differences may also be mirrored at the phenotypic level; the two studied animals were sampled because they differed in their growth rates relative to the amount of feed consumed (data not shown).

It would be very desirable to understand this difference in how feed is used for growth using predictive stoichiometric metabolic models (16,57–58). Unfortunately, such approaches do not currently scale to groups of hundreds or thousands of genomes nor is a sample of two animals sufficient for such a study. Note however, that as a matter of logic, two samples are fully sufficient for the twin conclusions of our study, which are essentially existence proofs. We have shown that it is possible to create microbial assemblages that differ more in taxonomy than in their detailed metabolism (e.g., that show differences not just in a handful of compounds but across the full metabolic network) and that the metabolic differences that are observed are inconsistent with the existence of only a single microbial metabolic network for a given environment. While more samples might refine our estimates of the frequency of these trends, they cannot dispute that such differences exist, either between taxonomy and metabolism or within metabolism. We cannot, at this point, assess ‘how’ these differences between animals are determined. Temporal variation (27) in the microbiome might imply that the differences observed at a single time point might not be maintained over the long term. However, the timescale of metabolism is necessarily fast relative to community turnover, so the existence of distinct community metabolic networks is still a relevant observation. Likewise, spatial variation within an animal is generally much lower than is variation between animals (59). The fact that the taxonomic profile of an animal's rumen can re-establish itself after ruminal transplant (60) also suggests that host/microbe interactions, including those across the network interface of Figure 2A, are partly responsible for animal-to-animal variation in ruminal ecosystems, rather than such variation being truly random.

The conclusion that these two steers are functionally similar in their metagenomic metabolic networks is hardly unexpected given the shared environment and diet. However, the fact that detectable differences are evident even under these rather controlled conditions, suggests another point: there is more than one way to build an ecosystem (61). This possibility manifests itself at two levels. First, the fact that the metabolic profiles of the two animals’ metagenomes are more similar than are their taxonomic profiles implies that different consortia of microbes can, at least in some circumstances, build the same metabolic structure in the ecosystem. As such, caution in the over-interpretation of taxonomic differences in metagenomic studies may be warranted. Second, even if we ignore taxonomy, the greater variability of the nodes farther from the host network in Figure 2A suggests that there may be several combinations of reactions and reaction frequencies that produce the same set of output compounds for the host, albeit presumably with subtle differences in their relative abundances.

This last result has an interesting linkage to one of the more surprising discoveries of systems biology: the highly redundant structure of metabolic networks (62,63). This redundant structure means that there are actually a vast number of potential metabolic networks that have very different enzymatic composition and yet manifest the same phenotypes (56). We therefore argue that results such as those above extend this principle of redundancy through multiple equivalent genotypes in single individuals to groups of organisms acting in an ecosystem. This link between population genomics and ecology has previously been noted in other contexts (64). It is worth considering whether this potential multiplicity of ecosystem metabolic networks could confound our attempts to qualitatively describe microbial ecosystems using taxonomic or gene counting approaches.

## ACCESSION NUMBER

Unfiltered metagenomic sequences are available from NCBI's short read archive under project accession number PRJNA291523.

## Supplementary Material

SUPPLEMENTARY DATA
