# Supplementary material for: The players may change but the game remains: network analyses of ruminal microbiomes suggest taxonomic differences mask functional similarity
Source: Nucleic Acids Res. 2015 Sep 29;43(20):9600–12. doi: 10.1093/nar/gkv973 (PMC4787786; doi:10.1093/nar/gkv973)
Supplement: SUPPLEMENTARY DATA [file supp_43_20_9600__index.html]

The players may change but the game remains: network analyses of ruminal microbiomes suggest taxonomic differences mask functional similarity — The players may change but the game remains: network analyses of ruminal microbiomes suggest taxonomic differences mask functional similarity — SUPPLEMENTARY DATA 

# The players may change but the game remains: network analyses of ruminal microbiomes suggest taxonomic differences mask functional similarity

## SUPPLEMENTARY DATA

- SUPPLEMENTARY DATA
- SUPPLEMENTARY DATA
