# Supplementary material for: The players may change but the game remains: network analyses of ruminal microbiomes suggest taxonomic differences mask functional similarity
Source: Nucleic Acids Res. 2015 Sep 29;43(20):9600–12. doi: 10.1093/nar/gkv973 (PMC4787786; doi:10.1093/nar/gkv973)
Supplement: SUPPLEMENTARY DATA [file supp_gkv973_nar-02187-n-2015-File005.docx]

**Supplemental Table 1:**

**Phyla identified by reads matching to 16S rDNA genes**

A total of 122 species-level OTUs were identified (*Materials and Methods*): below we summarize the distribution of these OTUs in phyla.

| **Phylum** | **Animal 1 reads matched** | **Animal 2 reads matched** |
| --- | --- | --- |
| Actinobacteria | 9 | 0 |
| Proteobacteria | 19 | 15 |
| Bacteroidetes/Chlorobigroup | 358 | 336 |
| Euryarchaeota | 11 | 16 |
| Synergistetes | 1 | 0 |
| Firmicutes | 1511 | 1851 |

**Supplemental Table 2:**

| **Interface Metabolites** | |
| --- | --- |
| **MetaCyc ID^a^** | **Common Name^b^** |
| 10-FORMYL-THF | 10-formyl-tetrahydrofolate |
| 16-HYDROXYPALMITATE | 16-hydroxypalmitate |
| 17-BETA-HYDROXY-5ALPHA-ANDROSTAN-3-O | 5-α-dihydrotestosterone |
| 2-DEOXYRIBOSE | deoxyribose |
| 2-KETOGLUTARATE | α-ketoglutarate |
| 2-METHYL-3-PHYTYL-14-NAPHTHOQUINONE | phylloquinone (Vitamin K_1)_ |
| 3-KETOBUTYRATE | acetoacetate |
| 4-AMINO-BUTYRATE | 4-aminobutanoate |
| 4-HYDROXYPHENYLACETATE | 4-hydroxyphenylacetate |
| 5-FORMYL-THF | 5-formyl-tetrahydrofolate |
| 5-HYDROXY-TRYPTOPHAN | 5-hydroxy-L-tryptophan |
| 5Z13E-15S-1115-DIHYDROXY-9-OXOPROS | prostaglandin E_2_ |
| 5Z13E-15S-9-ALPHA11-ALPHA15-TRIHY | prostaglandin F_2α_ |
| 5Z13E-15S-9-ALPHA15-DIHYDROXY-11-O | prostaglandin D_2_ |
| 5Z8Z11Z14Z17Z-EICOSAPENTAENOATE | eicosapentaenoate |
| ACET | acetate |
| ACETALD | acetaldehyde |
| ACETONE | acetone |
| ACETYLCHOLINE | acetylcholine |
| ADENINE | adenine |
| ADENOSINE | adenosine |
| ADENOSINE_DIPHOSPHATE_RIBOSE | ADP-D-ribose, ADP-ribose |
| ALDOSTERONE | aldosterone |
| ALPHA11-ALPHA-EPOXY-15-HYDROXYTHROMBA | thromboxane A_2_ |
| ALPHA-D-GALACTOSE | α-D-galactose |
| ALPHA-TOCOPHEROL | α-tocopherol |
| ANDROSTERONE | androsterone |
| ARABINOSE | α-L-arabinopyranose |
| ARACHIDIC_ACID | arachidate |
| ARACHIDONIC_ACID | arachidonate |
| ARG | arginine |
| ASCORBATE | ascorbate, L-ascorbate-6-phosphate |
| ASN | asparagine |
| B-ALANINE | β-alanine |
| BENZOATE | benzoate |
| BETA-D-FRUCTOSE | β-D-fructofuranose |
| BETAINE | glycine betaine |
| BETA-TOCOPHEROL | β-tocopherol |
| BILIRUBIN | bilirubin |
| BIOTIN | biotin |
| BUTYRIC_ACID | butanoate |
| CAMP | cyclic AMP |
| CARBON-MONOXIDE | carbon monoxide |
| CARNITINE | carnitine |
| CGMP | cyclic GMP |
| CH33ADO | 5'-deoxyadenosine |
| CHOCOLA_A | all-trans-retinyl palmitate (Vitamin A palmitate) |
| CHOLATE | cholate |
| CHOLESTEROL | cholesterol |
| CHOLINE | choline |
| CIT | citrate |
| COUMARIN | coumarin |
| CPD-10329 | α-L-fucose |
| CPD-10330 | α-D-ribofuranose |
| CPD-1112 | 4-pyridoxate |
| CPD-11408 | triiodothyronine sulfate |
| CPD-11877 | metanephrine |
| CPD-11878 | 3, 4-dihydroxyphenylglycol |
| CPD-12282 | ω-hydroxylaurate |
| CPD-12653 | stearidonate |
| CPD-12826 | folate |
| CPD-13030 | N,N-dihydroxy-L-phenylalanine |
| CPD-13038 | Xylose |
| CPD-13524 | all-trans retinol (Vitamin A) |
| CPD-13792 | docosapentaenoate |
| CPD-14268 | 15Z-tetracosenoate |
| CPD-195 | octanoate |
| CPD1F-129 | all-trans β-carotene |
| CPD1UA-5654 | Glucose |
| CPD-217 | D-ornithine |
| CPD-261 | (-)-perillyl alcohol |
| CPD-302 | D-aspartate |
| CPD-335 | (R)-3-hydroxybutanoate |
| CPD-3564 | L-2-hydroxybutyrate |
| CPD-367 | (2R)-3-sulfolactate |
| CPD-396 | 1-methylnicotinamide |
| CPD3DJ-11366 | sphingosine-1-phosphate |
| CPD-4592 | aflatoxin B_1_ |
| CPD-481 | sphingosyl-phosphorylcholine |
| CPD-4886 | L-limonene |
| CPD-4892 | α-pinene |
| CPD-611 | thiamin triphosphate |
| CPD-622 | (2S, 3S)-2-methylcitrate |
| CPD-649 | sphinganine 1-phosphate |
| CPD-7283 | taurochenodeoxycholate |
| CPD-7649 | dopamine 3-sulfate |
| CPD-7830 | heptadecanoate |
| CPD-7836 | myristate |
| CPD-8117 | γ-linolenate |
| CPD-8120 | di-homo-γ-linolenate |
| CPD-8186 | umbelliferone |
| CPD-8462 | pentadecanoate |
| CPD-882 | 11-cis-retinol |
| CPD-9247 | cis-vaccenate |
| CREATINE | creatine |
| CYS | cysteine |
| CYS-GLY | L-cysteinyl-glycine |
| CYSTINE | cystine |
| CYTIDINE | cytidine |
| CYTOSINE | cytosine |
| D-ALANINE | D-alanine |
| DEOXYADENOSINE | deoxyadenosine |
| DEOXYCYTIDINE | deoxycytidine |
| DEOXYGUANOSINE | deoxyguanosine |
| DEOXYINOSINE | deoxyinosine |
| DEOXYURIDINE | deoxyuridine |
| DIHYDROFOLATE | dihydrofolate |
| D-LACTATE | D-lactate |
| DOPAMINE | dopamine |
| D-PROLINE | proline |
| D-SERINE | D-serine |
| ESTRONE-SULFATE | estrone sulfate |
| ETOH | ethanol |
| FORMATE | formate |
| GAMA-TOCOPHEROL | γ-tocopherol |
| GLN | glutamine |
| GLT | L-glutamate |
| GLY | glycine |
| GLYCEROL | glycerol |
| GLYCOCHENODEOXYCHOLIC_ACID | glycochenodeoxycholate |
| GLYCOCHOLIC_ACID | glycocholate |
| GUANINE | guanine |
| GUANOSINE | guanosine |
| HCN | hydrogen cyanide |
| HIS | histidine |
| HISTAMINE | histamine |
| HOMO-SER | homoserine |
| HSCN | thiocyanate |
| HYPOXANTHINE | hypoxanthine |
| IDP | inosine diphosphate |
| ILE | isoleucine |
| IMP | inosine-5'-phosphate |
| INOSINE | inosine |
| ISOBUTYRATE | isobutyrate |
| ISOVALERATE | isovalerate |
| LACTOSE | lactose |
| L-ALPHA-ALANINE | alanine |
| L-ARABITOL | arabitol |
| L-ASPARTATE | L-aspartate |
| L-DEHYDRO-ASCORBATE | L-dehydro-ascorbate |
| L-EPINEPHRINE | L-epinephrine (R-adrenaline) |
| LEU | leucine |
| LEUKOTRIENE-C4 | leukotriene C4 |
| LINOLEIC_ACID | linoleate |
| LINOLENIC_ACID | α-linolenate |
| LIOTHYRONINE | 3, 5, 3’-triiodo-L-thyronine |
| LIPOIC-ACID | lipoate |
| L-LACTATE | L-lactate |
| L-THYROXINE | L-thyroxine (levothyroxine/T4) |
| LYS | lysine |
| MALTOSE | β-maltose |
| MALTOTRIOSE | maltotriose |
| MANNOSE | mannose |
| MET | methionine |
| METHYL-GLYOXAL | methylglyoxal |
| METOH | methanol |
| MYO-INOSITOL | myo-inositol |
| N-ACETYL-D-GLUCOSAMINE | N-acetyl-D-glucosamine |
| NAPHTHALENE | naphthalene |
| NIACINAMIDE | nicotinamide |
| NIACINE | nicotinate |
| NOREPINEPHRINE | noradrenaline |
| OLEATE-CPD | oleate |
| OXALATE | oxalate |
| PALMITATE | palmitate |
| PANTOTHENATE | pantothenate |
| PHE | phenylalanine |
| P-NITROPHENOL | 4-nitrophenol |
| PRO | proline |
| PROPIONATE | propanoate, propionate |
| PROTOHEME | protoheme IX (ferroprotoporphyrin IX) |
| PYRIDOXAL | pyridoxal |
| PYRIDOXAMINE | pyridoxamine |
| PYRIDOXINE | pyridoxine |
| PYRUVATE | pyruvate |
| RETINOATE | retinoic acid |
| RIBITOL | ribitol |
| RIBOFLAVIN | riboflavin |
| SARCOSINE | sarcosine |
| SER | serine |
| SEROTONIN | serotonin |
| STEARIC_ACID | stearate |
| SUC | succinate |
| SUCROSE | sucrose |
| TAGATOSE | keto-D-tagatose |
| TAURINE | taurine |
| TETRACOSANOATE | lignocerate |
| THF | tetrahydrofolate |
| THIAMINE | thiamin, thiamine |
| THIAMINE-P | thiamin-phosphate, thiamine-phosphate |
| THR | threonine |
| THYMIDINE | thymidine |
| THYMINE | thymine |
| TREHALOSE | α,α-trehalose |
| TRP | tryptophan |
| TYR | tyrosine |
| URACIL | uracil |
| URATE | urate |
| UREA | urea |
| URIDINE | uridine |
| VAL | valine |
| VITAMIN_D2 | Ergocalciferol (Vitamin D_2_) |
| VITAMIN_D3 | vitamin D_3_ |
| XYLITOL | xylitol |

a: Identifier in the MetaCyc database.

b: Inferred common name for the compound.

**Supplemental Table 3:**

Ingredients and nutrient composition of receiving diet.

| **Ingredient** |  |  |  | **%** |
| --- | --- | --- | --- | --- |
| Corn |  |  |  | 55.00 |
| Receiving Supplement |  |  |  | 35.00 |
| Hay |  |  |  | 10.00 |
|  |  |  |  |  |
| Nutrient Analysis^a^ |  |  |  |  |
| DM |  |  |  | 86.8 |
| CP^b^ |  |  |  | 20.85 |
|  |  |  |  |  |
| a: DM = percent dry matter of diet;  b: CP = percent crude protein of diet. | | | | |

| **Supplemental Table 4:**  Ingredients and nutrient composition of feedlot diet. | | | | | | | | | |  |
| --- | --- | --- | --- | --- | --- | --- | --- | --- | --- | --- |
| **Ingredient** | |  | |  | |  | | **%** | |  |
| Whole Corn | |  | |  | |  | | 72.35 | |  |
| Dried Distillers Grain | |  | |  | |  | | 15.00 | |  |
| Supplement | |  | |  | |  | | 12.65 | |  |
|  | |  | |  | |  | |  | |  |
| Nutrient Analysis^a^ | |  | |  | |  | |  | |  |
| DM | |  | |  | |  | | 86.28 | |  |
| CP | |  | |  | |  | | 18.12 | |  |
| a: DM = percent dry matter of diet;  b: CP = percent crude protein of diet.  **Supplemental Table 5:**  Body weights, dry matter feed intake, average daily body weight gain and residual feed intake values for the two animals analyzed for the ruminal metagenome. | | | | | | | | | |  |
| **Animal** | **IBW (kg)** | | **FBW (kg)** | | **DMI (kg)** | | **ADG (kg)** | | **RFI** | |
| 6W568 (1) | 301.82 | | 542.27 | | 2.00 | | -1.09 | | 9.88 | |
| 6W566 (2) | 279.55 | | 519.55 | | 2.00 | | 1.69 | | 12.16 | |

**Supplemental Table 6:**

Reactions from MetaCyc with the largest number of edges that had no reads mapped for either animal.

| MetaCyc Reaction Name^a^ | Currency cutoff^b^ | #Edges^c^ | Reaction equation^d^ |
| --- | --- | --- | --- |
| 2.4.1.217-RXN | 25 | 34 | GDP-mannose + 3-phospho-D-glycerate 🡪 GDP + H^+^+ alpha-D-mannosyl-3-phosphoglycerate |
| RXN0-5383 | 25 | 32 | Lipid A-core + H^+^ + undecaprenyl-diphosphate 🡪 di-trans,octa-cis-undecaprenyl phosphate + lipid A-core 1-diphosphate |
| O-PYROCATECHUATE-DECARBOXYLASE-RXN | 25 | 26 | H^+^ + 2-3-dihydroxybenzoate 🡪 CO_2_ + catechol |
| 1.14.12.1-RXN | 25 | 24 | H^+^ + NADH/NADPH + anthranilate +O_2_ 🡪 NAD^+^/NADP^+^ + CO_2_ + catechol + NH_3_ |
| SALICYLATE-1-MONOOXYGENASE-RXN | 25 | 24 | H^+^ + NADH + O_2_ + salicylate 🡪 H_2_O + CO_2_+ catechol+ NAD^+^ |
| RXN-12367 | 25 | 23 | α-Kdo-(2->4)-α-Kdo-(2->6)-lipid IV_A_ + 4-amino-4-deoxy-α-L-arabinopyranosyl *ditrans,octacis*-undecaprenyl phosphate 🡪 *di-trans,octa-cis*-undecaprenyl phosphate + 4'-α-L-Ara4*N*-α-Kdo-(2->4)-α-KDO-(2->6)-lipid IV_A_ |
| RXN-10444 | 25 | 23 | H^+^ + 2-iodobenzoate + O_2_ + NADH 🡪 iodide + CO_2_ + catechol + NAD^+^ |
| RXN-13418 | 25 | 22 | FMNH_2_ + isethionate + O_2_ 🡪 H_2_O + glycolaldehyde + H^+^ + FMN + SO_3_ |
| RXN-13221 | 25 | 22 | 2-deoxy-*scyllo*-inosamine + S-adenosylmethionine 🡪 H^+^ + methionine + 3-amino-2,3-dideoxy-*scyllo*-inosose + 5'-deoxyadenosine |
| RXN-12886 | 25 | 22 | thymine + FMNH_2_ + O_2_ 🡪 (*Z*)-2-methylureidoacrylate peracid + H^+^ + FMN |
|  |  |  |  |
| TEICHOICSYN1-RXN | 50 | 61 | UDP-N-acetyl-D-glucosamine + undecaprenyl-P + di-trans,octa-cis-undecaprenyl phosphate 🡪 acetyl-D-glucosaminyldiphospho-undecapre + UMP |
| RXN-6380 | 50 | 60 | UDP-N-acetyl-D-glucosamine + *trans,octacis*-decaprenyl phosphate 🡪 N-acetyl-α-D-glucosaminyl-diphospho-trans,octacis-decaprenol + UMP |
| RXN-12200 | 50 | 58 | H_2_O + CTP 🡪 P_i_ + H^+^ + CMP |
| RXN-8961 | 50 | 57 | (2*R*,3*S*)-β-methylmalyl-CoA 🡪 propionyl-CoA + glyoxylate |
| HYDGLUTSYN-RXN | 50 | 56 | H_2_O + glyoxylate + propionyl-CoA 🡪 H^+^ + CoA + 2-hydroxyglutaric acid |
| 2.7.8.17-RXN | 50 | 52 | UDP-N-acetyl-D-glucosamine + lysosomal-enzyme-D-mannose 🡪 lysosomal-enzyme-N-etcetera-mannose + UMP |
| RXN-4441 | 50 | 51 | P_i_ + trehalose 🡪 glucose-1-P + α-glucose |
| RXN0-986 | 50 | 50 | 1-ethyladenine + 2-ketoglutarate + O_2_ 🡪 succinate + acetaldehyde + adenine + CO_2_ |
| GLUCOSE-16-BISPHOSPHATE-SYNTHASE-RXN | 50 | 47 | glucose-1-P + 1,3-diphosphateglycerate 🡪 3-phosphoglycerate + α-glucose-1,6-bisphosphate + H^+^ |
| RXN-9508 | 50 | 46 | FMNH_2_ + nitrilotriacetate + O_2_ 🡪 H_2_O +  iminodiacetate + FMN + glyoxylate |
| CYTIDINEKIN-RXN | 100 | 101 | GTP + cytidine 🡪 GDP + H^+^ + CMP |
| RXN-14279 | 100 | 77 | malate + H^+^ + FAD^+^ 🡪 FADH_2_ + oxalacetic acid |
| RXN-2 | 100 | 75 | THF + H^+^ + vanillate + NADH + O_2_ 🡪 H_2_O + formaldehyde + 5-methyl-THF + 3-4-dihydroxybenzoate + NAD^+^ |
| RXN-9770 | 100 | 70 | methanesulfonate + NADH + O_2_ 🡪 H_2_O + SO_3_ + formaldehyde + NAD^+^ |
| RXN-14063 | 100 | 69 | GTP + adenosylcobinamide 🡪 adenosylcobinamide-P + H^+^ + GDP |
| PROPCOASYN-RXN | 100 | 68 | acrylyl-CoA + propionyl-CoA + FADH_2_ + ETF-Oxidized 🡪 propionyl-CoA + H^+^ + acrylyl-CoA + FAD^+^ + ETF-Reduced |
| METHANETHIOL-OXIDASE-RXN | 100 | 67 | H_2_O + O_2_ + methanethiol 🡪 HS + formaldehyde + H_2_O_2_ |
| RXN-9106 | 100 | 66 | solanesyl-pyrophosphate + delta3-isopentenyl-PP + farnesyl-PP 🡪 PP_I_ + all-*trans*-decaprenyl diphosphate |
| RXN-8081 | 100 | 65 | GTP + oxidized coenzyme F_420_^-1^ + glutamate 🡪 P_i_ + oxidized coenzyme F_420_^-2^ + GDP + H^+^ |

**a:** Name of the reaction in the MetaCyc framework.

**b:** Network used (e.g., *N_25_, N_50_* or *N_100_*)—see *Materials and Methods* for details.

**c:** Number of other reactions connected to this one in the metabolic network (see *Materials and Methods*).

**d:** Reaction formula as reported by MetaCyc.
