# Supplementary material for: The players may change but the game remains: network analyses of ruminal microbiomes suggest taxonomic differences mask functional similarity
Source: Nucleic Acids Res. 2015 Sep 29;43(20):9600–12. doi: 10.1093/nar/gkv973 (PMC4787786; doi:10.1093/nar/gkv973)
Supplement: SUPPLEMENTARY DATA [file supp_gkv973_nar-02187-n-2015-File006.pdf]

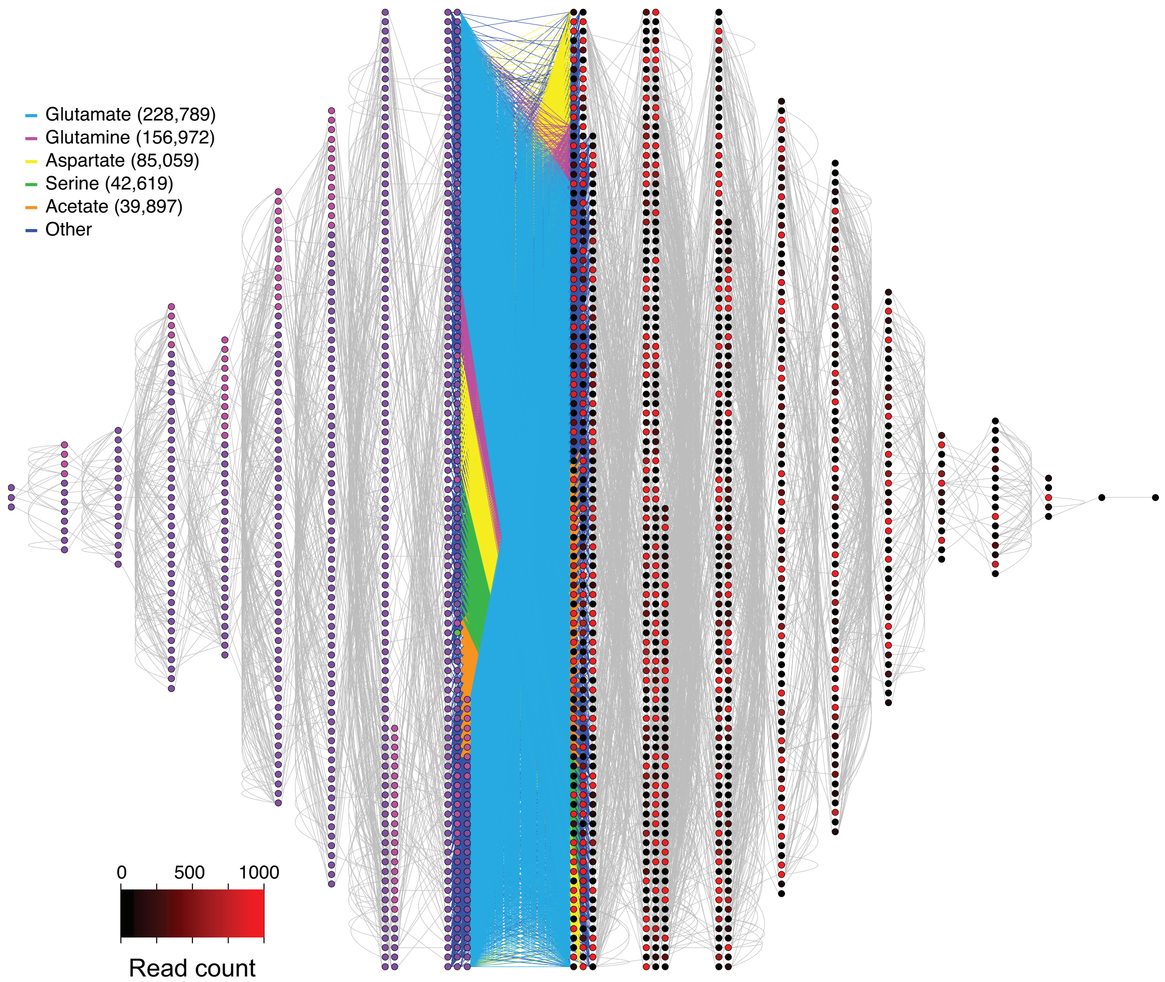

Supplemental Figure 1: Interface of the host (left) and microbial (right) metabolite networks. Nodes (circles) are reactions: two nodes are linked by an edge if they share a metabolite (grey lines, currency metabolites were excluded at a stringency of 25—e.g., N_{25}; see *Materials and Methods*). The interface of the two networks is illustrated in the center. The five most common interface metabolites are shown in color; all other interface metabolites are blue (set VFA+AA). Host/microbial reactions that employ an interface metabolite with the microbial network are shown as the two closest layers: subsequent layers are removed from this interface based on their nearest metabolites to that interface (*Results*). Microbial nodes colored by the total number of reads mapped across the two animals.

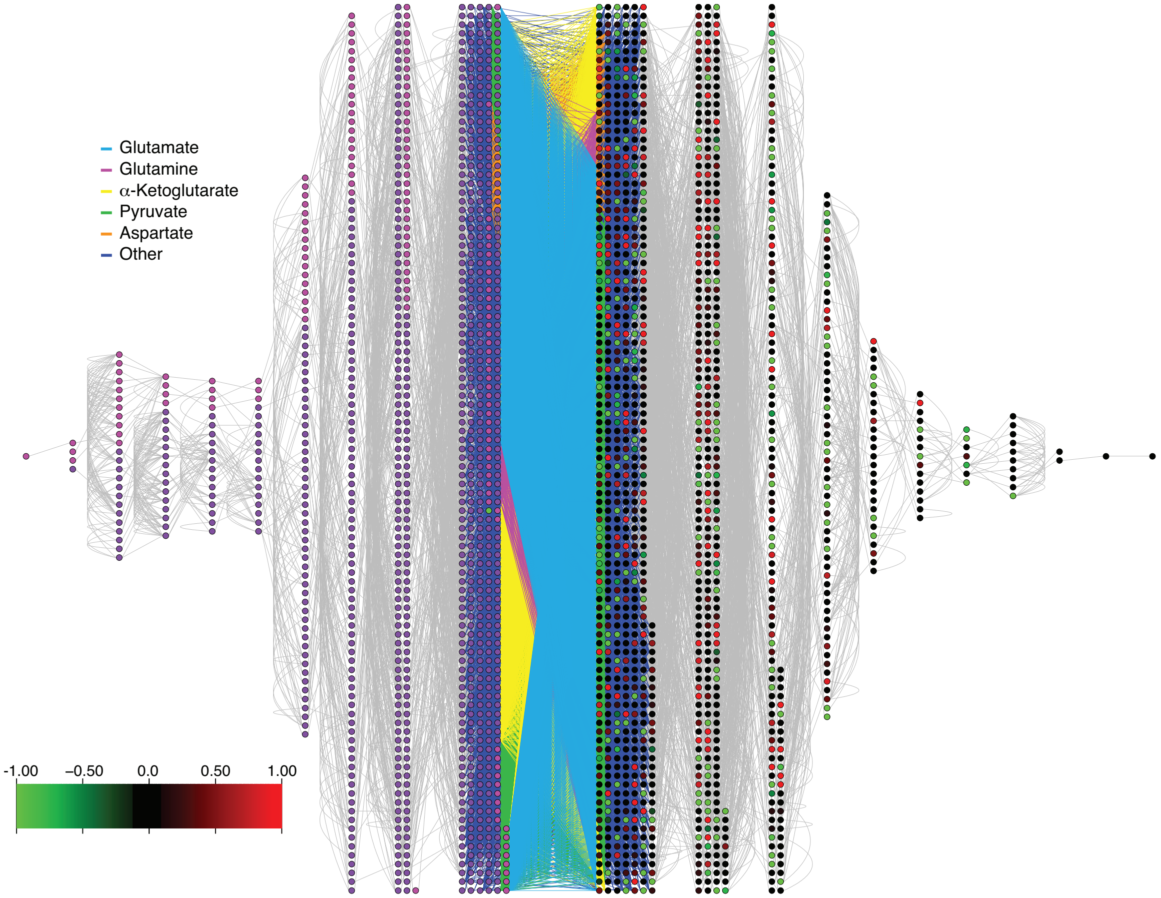

Supplemental Figure 2: Interface of the host (left) and microbial (right) metabolite networks when using the larger linking compound set VFA+AA+HUM. Nodes that share a metabolite are linked by edges. Currency metabolites were excluded at a stringency of 25—e.g., N_{25} (see *Materials and Methods*). The interface of the two networks is illustrated in the center. The five most common interface metabolites are shown in color; all other interface metabolites are blue. Host/microbial reactions that employ an interface metabolite with the microbial network are shown as the two closest layers. Microbial nodes are color-coded based on the $\log_2$ difference in the normalized number of read pairs mapped for animal 1 versus animal 2: thus green nodes represent over-abundance in animal 2 and red ones in animal 1. Only nodes with a binomial probability of $P \leq 0.01$ of having an equal numbers of reads are shown with fold-differences (all others shown in black).
